# Describing the end-of-life doula role and practices of care: perspectives from four countries

**DOI:** 10.1177/2632352420973226

**Published:** 2020-12-07

**Authors:** Marian Krawczyk, Merilynne Rush

**Affiliations:** Lord Kelvin Adam Smith Fellow, Glasgow End of Life Studies Group, School of Interdisciplinary Studies, University of Glasgow, Rutherford/McCowan Building, Bankend Road, Dumfries DG1 4ZL, UK; The Dying Year, Ann Arbor, MI, USA

**Keywords:** community care, death care, death doula, death midwife, dying, end-of-life care, end-of-life doula, hospice, palliative care

## Abstract

**Background:**

End-of-life doulas are emerging as a potentially important new form of community-based caregiving in the global North, yet we know little about this form of care. The aim of our study was to solicit the perspective of key stakeholders and early innovators in community-based end-of-life care about the development and practices of end-of-life doulas.

**Methods:**

We conducted 22 semi-structured interviews with participants in four countries where end-of-life doulas are most active: Australia, Canada, the United States, and the United Kingdom.

**Findings:**

This article focuses on participants’ description of the end-of-life doula role and attendant practices, and our findings provide the first detailed taxonomy of the end-of-life doula role and specific services on the basis of the perspective of subject experts in four countries. We situate our findings within literature on the professionalization of caregiving, with particular attention to nomenclature, role flexibility and boundary blurring, and explicit versus tacit knowledge. We also discuss the importance of jurisdictional considerations as the end-of-life doula movement develops.

**Discussion:**

We speculate that the end-of-life doula role is potentially experiencing common developmental antecedents similar to other now-professionalized forms of caregiving. Our findings contribute substantial new information to the small body of empirical research about the end-of-life doula role and practices, provide critical firsthand insight as the movement develops, and are the first research to explore end-of-life doulas from a comparative international perspective.

## Background/introduction

A new end-of-life care role is emerging in the global North. While there is no mutually agreed descriptor of this role, the appellation ‘end-of-life doula’ is increasingly used as an umbrella term to identify lay people, primarily women, who provide a diversity of nonmedical supports—social, emotional, practical, and spiritual—for people nearing the end of life, including those close to them. The term ‘doula’ derives from the Greek word meaning female slave or servant, and was popularized by the natural birth movement in the 1970s to describe lay-trained women providing nonmedical assistance during and after pregnancy.^1,2^ End-of-life doulas (EOLDs) explicitly draw from this nomenclature and model, providing informed companionship and resources before, during, and after death. In some regions, support may include after-death care of the body and funeral planning education or services. Practitioners also host community education and social events such as advance care planning workshops and Death Cafes. Collectively, EOLDs frame their work as both a ‘reclaiming’ of community tradition and as a new quasi-professional role within rapidly changing social and health care environments. EOLDs and their advocates argue that this role holds the potential to radically improve end-of-life care through empowering individuals, developing ‘compassionate communities’, and reducing burden on health care systems.^1–3^

Laywomen have long engaged in community end-of-life and death care, well before the concept of EOLDs emerged.^4,5^ The first formal use of ‘doula’ to describe a specific kind of end-of-life accompaniment was employed by the ‘Doula to Accompany and Comfort Program’, a grassroots volunteer-driven model launched in 2001 through the New York University Medical Centre’s Department of Social Services to focus on the social, psychological, and spiritual needs of individuals at risk of isolation during the dying process.^6,7^ While similar EOLD volunteer models have been developed elsewhere, particularly within hospice and palliative care programs,^7–9^ overall these institutional programs remain relatively rare. The last few years have instead evidenced the EOLD role developing primarily as an independent community-based role.

EOLDs have captured widespread attention in the global North. In-depth features about their work have become common media content, with numerous articles appearing across mainstream platforms such as the *BBC, The Guardian, Huffington Post*, and *The New York Times*. Interest is particularly strong in Australia, Canada, the United Kingdom, and the United States, and these countries are where the development of an EOLD ‘movement’ has been most evident. Each of these countries has seen a rapid proliferation of training programs offered by entrepreneurial individuals, nonprofit organizations, and higher education institutions. Practitioners are also self-organizing into associations and developing core competencies and practice guidelines, as well as pursuing accreditation pathways.^10,11^

EOLDs evidence a potentially important new response to changing norms, desires, and concerns about the end of life and end-of-life care. At the same time, this role is still developing and in flux. Given their grassroots history, practitioners do not adhere to a mutually agreed-upon set of practice standards or scope of practice, as even the name of their role is a source of difference and debate. While ‘end-of-life doula’ and ‘death doula’ are becoming the most common umbrella terms for a specific set of practical, emotional, and spiritual support services, these and other titles encompass a range of predeath and postdeath services that can vary widely between individual practitioners, trainers, regions, and countries.^2,3^ Alternatively, different practitioners may use different titles and yet provide the same services.

EOLDs operate at the edge of formal health care systems and, currently, on the margins of academic research. In 2016, one prominent UK EOLD trainer estimated that there were 100 practitioners in the United Kingdom,^12^ and in 2018, a Canadian practitioner estimated 40 practitioners in the province of Saskatchewan alone.^13^ There is a small but growing body of literature on the topic,^1,2,3,7,14,15^ including three academic dissertations.^6,13,16^ Of particular note are Rawlings and colleagues’^14^ three Australian publications: a systematic review of literature in 2018 describing the role/work of death doulas and death midwives, followed by the results of their online survey with people identifying as death doulas^2^ and interviews with a subset of those survey participants.^15^ Their review found only a handful of publications, along with substantive variation in the way the role was described and understood within the literature. Their survey results ‘corroborate strongly’ with their systematic review, finding significant uncertainty among respondents about whether all doulas offer the same services, as well as inconsistencies in how the role was described and enacted. The authors conclude that this evidences ‘a generalized confusion within the industry’ (p. 19).^2^ This study offers important insights about the ways in which the death doula role is conceptualized and practiced. It also has serious limitations, with half of the surveys only partially completed, and some respondents either not practicing as a death doula or conflating it with other health care roles. Finally, their interview study highlights the simultaneous complementarity and tensions between the services EOLDs can provide and palliative/hospice care, as well as the ongoing heterogeneity of individual doula practices.^15^

The aim of our study was to learn more about the development and practices of EOLDs from the perspective of key stakeholders and early innovators in community-based end-of-life care.^1^ We also wanted to better understand key issues that may both support and challenge the future development of this role. Empirically, this study is based on semi-structured interviews with 22 participants in four countries: Australia, Canada, the United States, and the United Kingdom. In this article, we focus on the ways in which participants describe the EOLD role and scope of practice, and we compare key jurisdictional variations shaping country-specific practice. Our research adds substantial new knowledge to the small body of empirical research on death doulas, death midwives, and EOLDs, and offers the first international comparative insights into the topic.

## Methods

Participants were recruited through a combination of purposive and convenience sampling. We chose countries for recruitment on the basis of an Internet search of EOLD organizations and training programs, a review of the literature, a media scan, and informal discussions with subject experts. Initial participants were approached on the basis of these country-specific findings and the authors’ combined knowledge of the subject area. MK has extensive academic research experience in palliative and end-of-life care in Canada and the United Kingdom; MR is a well-known EOLD practitioner and trainer in the United States, has been involved in the home funeral and green burial movement, and has held several high-profile positions within national EOLD organizations. Participants were also asked who else we should speak with; we prioritized those mentioned more than once and those with the most experience in the field. Our sampling strategy was to achieve a broad overview of key issues shaping the EOLD movement each country, rather than seeking saturation.

In total, we approached 30 people; two declined and six did not return our inquiry after a follow-up email. We conducted five interviews with participants in both Australia and the United Kingdom, and six interviews with participants in both Canada and the United States. Interview questions focused on (1) participants’ personal and professional backgrounds relevant to the EOLD role, (2) describing the EOLD role and specific practices, (3) why the growth of interest in EOLDs, and (4) current and future considerations in practice and training. Interviews ranged from 41 to 117 min, with an average length of 82 min. One interview was cut short, and we were unable to successfully reschedule. One participant was employed in an academic administrative role, and as she did not identify as a practitioner or direct trainer, some of her responses are excluded below where not relevant.

Both authors equally functioned as interviewers, although MR did not conduct any interviews with anyone she knew or worked with. We used Zoom as our interview platform. All participants provided written informed consent. Ethics was approved by the University of Glasgow (400180148). Interviews were professionally transcribed verbatim. Transcripts were then anonymized by MK, who also conducted the initial categorizing of responses in NVivo 11, a qualitative analysis software program from QSR International. Both authors then each separately blind coded eight transcripts (two from each country) to develop key themes. Afterwards we met for a 2-day intensive analysis session to compare notes, reconcile differences, and develop a detailed codebook. We then divided the remaining transcripts and coded them independently using the codebook as a guide, and met biweekly to discuss results and further refine the codebook. Finally, MK reviewed all transcripts in their entirety a final time to ensure that developed themes represented a ‘whole picture’ approach to the respondents’ perspectives. We contextualize our analysis with direct quotes marked by a transcript number which enables us to evidence the diversity of perspectives while ensuring anonymity.

We used an abductive and iterative approach to analysis^17^ and employed a narrative social constructionist framework.^18^ This entailed ‘tacking’ back and forth between developing our analysis based on preexisting interests and interview questions, and being open to new or anomalous observations that did not fit existing theories, allowing for conceptual innovation. Our analytic robustness was further strengthened by the authors’ differing backgrounds, and at times perspectives.

## Participant characteristics

All participants (bar one) identified as a practitioner, trainer, and/or educator in community-based end-of-life care. Twenty respondents reported providing some form of direct community-based end-of-life care (including after-death care) outside of an existing professional health or social care role. Fourteen respondents stated they provided both paid and volunteer services, three participants stated they provided services on a volunteer-only basis, and one said they let their clients decide how to reimburse them—if at all. There are missing data about reimbursement for two participants. Two thirds of respondents (14 of 21) currently or in the past have provided some form of training courses or programs.

Approximately half (11 of 21) used ‘end-of-life doula’ as a practice title; the others did not, preferring other descriptors. Participants were also divided on the basis of whether they employed more than one title to describe their practice, with roughly half using a single descriptor and half using more than one. Of those who used only one, nine solely used the title ‘end-of-life doula’, one participant only used ‘death midwife’, and another only ‘soul midwife’. See Table 1 for more detail about titles used.

**Table 1.** Practice titles used (*n* = 21).

Role descriptors used	Number of times used by participants
End-of-life doula	11
Death midwife	4
Death doula	3
End-of-life consultant	3
(Ideally) Choose not to identify	3
End-of-life educator/education	2
Death caring/carer	2
Funeral director/home funeral guide	2^a^
Death walker	1
Soul midwife	1
End-of-life midwife	1
End-of-life guide	1
End-of-life coaching	1
Thanadoula	1
End-of-life care doula	1
Circle of life practitioner	1
Death care educator	1
End-of-life practitioner	1

a These respondents also provided predeath care and/or education.

Those who used the term EOLD did so to highlight the connection with, and similarities to, birth doulas, including the natural birth movement as a whole; to highlight the similarities between pregnancy/birth and dying/death; to champion the generalized benefits of the doula role through all major life transitions; to distance themselves from the descriptor ‘death doulas’ (both because they felt it did not fully describe the temporal scope of their practices, as well as a perception of public dislike for the word ‘death’); due to the term’s increasing popularity; and/or because it was the role descriptor they first encountered for this set of care and support practices. The connection between birth and death was a particularly common reason given for using the term ‘doula’, and is illustrated in the following anecdote recounting a staged public conversation between participant and a birth doula at a national doula conference.

And she goes, ‘Oh my God, I’ve just found out I’m pregnant’. And I go, ‘Oh my God, I’ve just found I’m dying’. And she says, ‘Who will I tell first?’ And I go, ‘Who will I tell first?’ And she goes, ‘Will I do it at home, or will I do it in hospital?’ I go, ‘Will I do it at home, or will I do it in hospital?’ She goes, ‘Will I take drugs, or will I do it naturally?’ You get the point, right. And we did 20 separate things. And it was just mirrored, it was exactly the same, it was so powerful, people were just sitting there … So, we coined the phrase, a doula, is a doula, is a doula. (T1)

Interestingly, four of our participants reported they independently began using the term EOLD without knowing of any other previous use of the term.

Reasons for not using the EOLD title included belief that the descriptor does not represent the centrality of after-death work; having started practicing before the descriptor became popular; the term not reflecting practitioners’ own unique philosophies and practices, and/or challenges of using the term within medical settings and among health care professionals.

I am really wary about what I call myself because … it’s more about a way of working rather than a title. Like should a death doula be that point up until the point of death and then should there be another person? Or should there … personally I prefer the word midwife … I think more people are actually familiar with the role of a midwife, you know, and it carries a certain … an expectation really. And that I guess [is that] a midwife is somebody that is with you all the way through. (T21)

I’m finding that within the aged care space and within the medical practitioner space that an end of life consultant or end of life educator, that those particular terms are really assisting me to overcome some of the immediate barriers that I was finding around the use of the word ‘doula’. (T12)

Respondents were asked how long they had been involved in providing, training, and/or developing services in community-based end-of-life and after-death care. Responses ranged from 3 to 35 years. Approximately 50% of participants had 10 years or more of experience; 30% reported between 4 and 7 years of experience, and 20% had 3 years or less experience. Respondents in this last category had been named by more experienced participants as important new practitioners, trainers, and/or educators to engage.

Virtually all participants (20 of 21) reported professional health care, social care, and/or educational backgrounds such as nursing and social work practice, employment as midwife and/or birth doulas, relevant academic degrees (e.g. psychology, sociology), and training in mental health, Chinese medicine, and hospice volunteering. Participants also reported nonrelated university degrees, training in arts and music therapies, and many reported practicing complimentary, alternative, and holistic therapies such as Reiki and aromatherapy. Two participants also had a background in home funerals. A majority (17 of 21) mentioned having studied with noted community-based end-of-life and after-death care practitioners (both nationally and internationally) while developing their own practice, often naming other participants in this study.

We asked participants what drew them to this type of work. Slightly more than half (12 of 21) referenced a significant death of someone close: a parent, grandparent, or friend, and they often had been a caregiver of that person. On an aggregate level, this impetus for further interest did not seem to necessarily correspond to these being ‘good’ or ‘bad’ deaths, but rather to the experience of accompaniment itself. Other common reasons were ongoing personal interest and/or work experiences in end-of-life care (8 of 21), involvement with the home birth and/or home death movement (6 of 21), always having been interested in the end of life (5 of 21), and due to growing interest in and open conversations about dying and death within their communities and among the general public (3 of 21). Participants often referenced more than one reason.

## Describing the EOLD role

Participants were asked to describe the EOLD role. Initial responses varied widely as many identified the challenges of trying to encompass the diversity and idiosyncrasy of individual practitioners, as well as the uniqueness of each doula–client relationship. Further, some participants answered based primarily on their own practices. Finally, some participants did not identify as an EOLD, even if the services they provided overlapped substantially, or completely, with what others identified as EOLD services. In these instances, we asked respondents for their understanding of the role even if they did not identify as an EOLD. Responses therefore often started with qualifying statements such as ‘I don’t know if there’s one answer’ (T14), and ‘The general [definition] is a bit hard, I think because it’s very broad’ (T9).

Well, [a definition] it’s difficult to pin it right down. The way we talk about that in the training is to try and invite people constantly to come back to simplicity and the role fundamentally is a companionship role that there’s a commitment to consistent presence and that presence needs to be very flexible and responsive, in the moment … It’s a bit woolly in a way but the wooliness is out of necessity. (T21)

Others evidenced little or no hesitation in describing the role. Regardless of response, however, when interviews were taken as a whole, there was significant agreement about key characteristics of the role across all participants. The most common words or category of words used to describe the role were *support* (across practical, emotional, physical, and spiritual domains), *educate* (providing resources, information, and teaching practical skills; ‘naturalizing’ the dying process), and *empower* (increasing individuals’ and families’ capacity to make end-of-life decisions and/or provide care; enhancing the larger community’s ability to ‘reclaim’ dying). A secondary group of common descriptors included *companion and presence* (to listen, take time and ‘hold space’), *advocate and champion* (enhance the voice of individuals and families), *coordinate, collaborate, and facilitate* (linking information and services within and between families, communities, health care systems), *mediate and guide* (navigate through myriad end-of-life processes; relationship and communication issues), and *assess and plan* (evaluating client and family needs; structured conversations about advance care and legal planning; after-death wishes). Finally, for a significant minority, the role also included *after-death care and funereal work* (providing hands-on services or instruction to wash and dress the body, how to keep the body at home, transportation to funeral home, and/or home funerals, celebrancy, and burial). Many also spoke about the EOLD role, in discussion with clients’ desires, as facilitating particular *rituals for meaning-making* (legacy work, prayer, use of candles and music at bedside) and to *enhance death literacy* in the wider community (informal conversations, public speaking, free advance care planning workshops, facilitating death cafes).^2^ Table 2 aggregates these descriptors.

**Table 2.** Most common descriptors of the EOLD role.

Primary descriptors	Secondary descriptors	Tertiary descriptors
Support	Companion and presence	After-death care and funeral work
Educate	Advocate and champion	Rituals for meaning-making
Empower	Coordinate, collaborate and facilitate	Enhanced community death literacy
	Mediate and guide	
	Assess and plan	

EOLD, end-of-life doula.

Respondents identified the benefits of the EOLD role across a broad spectrum of people, care locations, and time. This included the person with advanced illness, their families, their social networks, their health care providers (particularly hospice and palliative care teams), and the larger community. While most respondents stated that EOLDs predominantly work with individuals living at home, many also discussed the relevance of the role within hospice, hospital, and long-term care facilities, as well as informal care occurring through spontaneous conversations with community members and friends. Most participants identified the benefits of having an EOLD before, during, and after death, including at diagnosis, while living with illness, while receiving palliative care, during active dying, immediately after death, at the funeral (if person or family requests), and during the first stages of bereavement. Given that most respondents described their work as an in-person process, few discussed other modalities of practice, although some mentioned WhatsApp and telephone, and many framed their participation in various public events and in the media as advocacy and therefore a type of community care.

## Describing EOLD services

Similar to participants’ general description of the EOLD role, descriptions of specific or core services that ‘should’ or ‘should not’ be provided in this social model of care evidenced a heterogeneity of perspectives.

### Are there specific services that EOLDs should provide?

Initial responses to this question were often generalized through reference to the above role descriptors (e.g. ‘to support’) rather than any concrete services or specific tasks. Further discussion was again usually qualified by acknowledging the individuality of practitioners’ backgrounds, skills, and interests, as well as the singular relationship between doulas and each client. As a result, respondents primarily detailed the services they themselves provided or offered in their training, or that hypothetically ‘could’ be provided, dependent on client wishes. This flexible perspective is summed up in the words of one respondent who stated ‘… we are the human Swiss Army knife because we can just do anything as long as it’s legal and ethical’ (T17). Others compared the potential smorgasbord of EOLD services as similar to a ‘stage manager’, ‘event planner’, or ‘wedding planner’.

Again, however, when looking across the interviews as a whole, there were significant commonalities in perspectives. We organized the most frequently referenced ‘types’ of services under the categories of *coordination and navigation, emotional and spiritual support*, and *death literacy and information transfer*. A close secondary category, based on the number of mentions, included *companionship and presence, basic practical and personal care*, and *after-death care services*. Table 3 enumerates EOLD-specific tasks within the above taxonomy of services.^3^ This table is neither prescriptive nor definitive, and does not necessarily indicate agreement among practitioners; rather, it is offered to highlight both the diversity and scope of services and specific tasks specifically mentioned within the interviews.

**Table 3.** Types of EOLD services and specific tasks.

Type of services	Specific tasks
**Coordination and navigation**	• Advance care planning, including organ donation • Liaise with hospice, other health care workers, and other community services (e.g. home support) • Facilitate legal paperwork in conjunction with relevant professionals (e.g. advance directives, power of attorney, estate planning) • Referral to community resources (practical, emotional, spiritual) • Organize informal care networks • Keep family members informed • Coordinate family and friend visits • Develop ‘departure directions’ including vigil planning, and/or assist in funeral coordination
**Emotional and spiritual support**	• Ask questions to understand emotions • Meaning-structured life review sessions • Discuss values and desires; spiritual beliefs • Legacy work (e.g. narrative work, assisting people to write their life stories; write/record last messages) • Talk with children • Visualization and guided imagery; tapping; breath work and touch; and energy work (e.g. Reiki) • Music therapy (e.g. singing, playing instruments, listening to favorite songs) • Design and conduct living funerals • Generalized predeath and postdeath bereavement support (e.g. talking, listening, and giving resources)
**Death literacy and information transfer**	• ‘Help explain diagnosis and treatment’ (e.g. help client get needed information) • ‘Normalize end of life’; provide practical information about what to expect; and explain common signs and symptoms at the end of life • Provide practical information about basic end-of-life care for family/friends • Inform family/friends about what they can do during active dying and after death (e.g. get into bed with the person, tell stories, washing the body) • Inform about funeral options, including home funeral options (e.g. keeping and transporting the body, after-death documentation) • Communicate regional legalities related to death care • Preview of crematorium (if desired) • Community work and advocacy (e.g. advance care planning workshops, death cafes, public speaking) • Informal/spontaneous conversations about end-of-life planning and care with friends, family, and community members
**Companionship and presence**	• Listen (e.g. client reminiscing) • Unstructured conversation • Read together (e.g. scripture, prayer, favorite books/poetry) • Hospital appointment accompaniment (if necessary) • Hospital/hospice in-patient visiting • Vigil (being present during the active dying phase, including talking and/or singing even if unresponsive) • Create bedside rituals (e.g. lighting a candle, blessings)
**Basic practical and personal care**	• Regular assessment of pain and symptoms • Respite for family members • Assorted housework (e.g. helping with meals, changing sheets, hanging out with kids, shopping, dog walking, hedge cutting) • Help wash and toilet; emergency assistance (e.g. ‘wiping bum if needed’) • Light massage and/or use of essential oils for pain and symptom management • Vigil care (e.g. mouth care, repositioning in bed, changing bedsheets, applying cool compress) • Give medication if trained by family^a^
**After-death care**	• Body care (e.g. washing body, ceremonial or entire cleansing) either alone or instructing family • Assist in keeping the body at home after death; assistance with after-death paperwork • Funeral celebrant • Funeral attendant (if requested) • Check-in with client’s family/friends after period of time (including bereavement support)

EOLD, end-of-life doula.

a Only one respondent gave this answer.

Overall, participants asserted that any EOLD process (i.e. services and individual tasks) should facilitate an outcome of ‘empowerment’. The concept of empowerment was employed in three overlapping ways. On the individual micro-level, it referenced the ways in which EOLD services are a form of holistic personalized care empowering clients and their families through enhancing their quality of (remaining) life and ability to make informed decisions. On the meso-level, empowerment through EOLD services is based on a community activist role, facilitating the collective capacity to develop local end-of-life care resources and skills, eventually making EOLD redundant. Finally, on the broader macro-level, respondents framed EOLD services as empowering (and reflecting) a broader cultural shift to ‘reclaim’ dying and death, with doulas themselves as vanguards and ideological change agents challenging normative biomedical framing of death as primarily a medical event.

However, there were significant tensions evident about how best to achieve these outcomes. For many, a key concern was how to provide services without creating new forms of expertise and dependence, thereby recreating the very knowledge and care infrastructures seen to have directly led to the doula movement in the first place.

For me it’s not about providing resources to people. It’s about resourcing people. And to me resourcing people means that by the time that I leave that family or I leave that person, that they then have the skills in place that they can take back further and that they can go on and find their own resources when I’m not there, because otherwise I’m just creating dependency. So my struggle with the terms ‘consultant’ and ‘educator’ are that in some ways they sound as though I am the holder of that knowledge. Therefore come to me and I can provide that to you. And that isn’t what I do. I’m not providing something to someone. I’m encouraging them to develop the skills so that they can make the choices and have the power, feel this … empowered to have those choices implemented. (T11)

Yet as previously noted, many respondents also identified that continuous presence and hands-on care were key aspects of their work. This simultaneous both/and nature of the role was often conceptualized as ‘walking alongside’, doing *with* (individual, family, community) where possible, and doing *for* when necessary.

Respondents also spontaneously articulated significant personal qualities or characteristics—conceptualized by several as the ‘doula heart’—needed for this work. Particularly important were the benefits of having a broad range of life experiences, specifically with the end of life, as well as significant insight about oneself and one’s past experiences. This resonates with Fukuzawa and Kondo’s understanding of original definition of doula to mean an experienced ‘mature’ woman (p. 617).^1^ For our interview participants, this maturity was key to developing holistic, flexible, and compassionate therapeutic relationships with clear boundaries. Specific aspects of these relationships included developing self-care routines, not pushing any services the client did not want, carrying expectations of how people are ‘supposed’ to behave at the end of life, imposing their own agenda (including spiritual agenda), becoming overinvolved or creating dependency, speaking ‘for’ the client or attempting to ‘fix’ complex family dynamics, or promising a good death.

### Are there specific services that EOLDs should not provide?

On an aggregate level, responses to this question generally evidenced the perspective that—in words of one participant—EOLDs ‘should not provide any service which falls under the jurisdiction of another licensed professional’ (T5). For many respondents, however, there was also an accompanying awareness that clear delineation of services under different professional umbrellas can be challenging in practice. Responses about what services EOLDs should not provide were particularly diverse in relation to emotional support, basic personal and practical care, and after-death services.

The overwhelming majority of respondents were clear that EOLDs do not provide medical care or legal advice, even as some acknowledged that the ‘lines’ between EOLD services and medical care are easily blurred.

To me, an end of life doula is a non-medical role, and I think it’s important we keep that distinction. I think it gets a very messy, blurry line there, and I think there’s a lot of regulation around medical and nursing, and I think that’s accurate. So, I don’t believe we should provide those. (T1)

Several participants were careful to delineate that medical care could be provided by an EOLD if they were also a licensed/certified health care professional, but that it must be clear those particular services were being done under that role’s scope of practice and regulatory authority rather than as an EOLD role. Others highlighted that their capacity to bridge different forms of care, including basic medical care, was a desired service feature for many clients, and/or an integral to EOLD philosophy and practices of care.

Me, I also feel helping with practical tasks which may include turning the person and stuff like that. I know there’s a lot [of conversation] right now … [that] doulas don’t do medical. Okay, well define medical. If medical is giving medication, okay, if you don’t want to give it, that’s fine. But it’s my opinion that if the family teaches you, how they want you to feed, bathe, change, turn, give medication, then you’re under the family’s teaching. And to me that’s okay … my belief about the true value of a doula is about respite for the family also. Not just coming, going, as a consultant. That’s [home hospice]. A doula’s spending time, we’re the spending the time that hospice can’t spend. So that means we’re doing a lot more than consulting. (T6)

As evidenced in the previous quote and detailed in Table 3, many participants felt practical and personal care—such as basic physical care and supporting activities of daily living, including household activities—were key services desired by many clients, and foundational to the EOLD role. Others expressed reservations, and the need for formal safeguards, when providing any type of practical or personal care, including respite.

We also caution doulas just to be aware of if you’re providing respite and no one else is around, you just need to have your contract well-developed, you need to be carrying insurance, and you need to know what their plan is, so if this happens, who do you call? Who do you reach out to? What are you supposed to do if something drastically shifts in terms of the health of your client? (T8)

Some stated that while they would provide emergency assistance (e.g. client incontinence), they did not want to take care tasks away from the family or, alternatively, veer into the role of a home care provider. Yet as evidenced earlier, practical and personal care services were also commonly employed in defining the EOLD role, and reference to them can be seen in many public definitions.^10–12^ One practitioner detailed how she attempts to negotiate this ambivalence:

So I do a 20 minute rule for myself. What can I get done in 20 minutes before I leave? Whatever they need before I leave to make their evening, their day, whatever, look a little easier I will do, but I’m not going to clean a cat litter box or shovel snow. But why wouldn’t I shovel snow if I know [the client] has to leave? It’s so hard, because you’re there to serve them, you’re there to care for them, you’re there to do the things that aren’t getting done for them, but at what level? (T19)

The challenges of blurred boundaries were also particularly evident in discussions of emotional support, including bereavement follow-up. While this form of support was articulated as a core aspect of EOLD services, many participants also expressed concerns about EOLDs being able to discern the dividing line between ‘supportive’ services and formal counseling.

Considering family dynamics and considering that when we’re in really raw grief, and if our mourning process is troubled or stymied or mis-framed or misdirected, that can go wrong really quickly. And I think it could be a very dangerous space to offer something therapeutic in terms of emotional support for grief and loss. Unless the practitioner is, a) really well supported and really well boundaried, and b) really well trained. (T3)

As highlighted in the above quote, some respondents evidenced concerns about the EOLD role after the death of the client. Further, while many participants identified after-death services such as washing and dressing the body (or providing guidance for family on how to do this), funeral celebrancy (if trained), and bereavement follow-up as key services available and central to the care role, some acknowledged that not all doulas want to provide these services.

I have a colleague who is only interested in going into the hospital, being with the person who is at end of life or dying. She doesn’t interact with family and friends. And when someone dies, that’s it. She doesn’t do after-death support at all … So I think as long as there’s very clear communication about what it is you do as an individual … From my perspective and saying well, this is all core doula business. Because I would argue that after-death care is core doula business. But not all end-of-life doulas are going to want to go there. (T5)

This diversity of perspectives about after-death care can also, in part, be explained by differences in practice titles, as all participants who used the title death midwife provided these services and identified them as core to their role. Differences in after-death care also require a broader understanding of jurisdictional and international differences in end-of-life care.

## Jurisdictional and international differences

Back in the day it was just like we were called to do this kind of work and we find ourselves now in the middle of this beautiful movement but, you know, ten years of experience, you know, is ten years of experience. So it’s so funny because you never would think to be finding yourself at this place … And here we are now, you know, in a worldwide movement, which is incredible. (T10)

Almost all respondents mentioned training and/or working with other practitioners, with approximately half mentioning those in other countries, indicating a great deal of awareness and collaboration internationally. Despite the socioeconomic similarities between the countries where EOLDs are most active, however, there are also key jurisdictional and international differences shaping community-based innovations in end-of-life care, most prominently health insurance coverage, hospice palliative care models, assisted dying legislation, and funeral industry regulations.

Australia, Canada, and United Kingdom all have variants of a publicly funded model of universal health care, using a mix of nonprofit and for-profit insurance provided by the state, through employers, and/or through individual policies. How the funds are spent is dependent on provincial, state, and health regions. Currently ~47% of Australians buy additional private complementary (e.g. access to noncovered and/or private services and benefits) and supplementary coverage (e.g. increased choice, faster access for nonemergency services), ~67% Canadians buy additional private complementary coverage, and ~11% of people in the United Kingdom do so.^19^ The United States, however, follows a hybrid private/public funding model, with private primary insurance covering ~66% of the population.^19^ As a result, some respondents identified the ways in which EOLD services may eventually be reimbursable within specific jurisdictions which will be shaped not only by the professionalization of their services and integration into health systems but also by regional differences and models of health care insurance coverage such as the development of insurance billing codes, and/or regional initiatives for patients to receive direct funds from local health authorities to purchase their own discretionary health care services.

While Australia, Canada, the United Kingdom, and the United States have all integrated hospice palliative care within mainstream health care services, how these services are provided varies within and between countries. Several Australian and Canadian respondents stated that most forms of hospice palliative care in their respective countries are institutionally based, and identified a lack of community services outside of densely populated areas.^13^ These particular challenges were less referenced by UK participants where there is significant third sector involvement in end-of-life home care, such as Marie Curie and Macmillian Cancer Support, although several participants noted the resource and support limitations of these services. The United States has both nonprofit and for-profit hospice care, and several American respondents expressed concern about inconsistent levels of good hospice care and institutional health care fraud and highlighted how large for-profit corporations are buying up hospices and/or using questionable marketing practices. At the same time, the United States is currently the only country which has any (volunteer) EOLD programs embedded within hospices and hospitals. Regardless of the country, the majority of respondents identified the role EOLDs can play as an integral part of the client’s hospice and palliative care team, and/or how EOLDs can assist in alleviating the time and resource gaps faced by health care providers.

Assisted dying is legal in Canada, in several US states, and has recently been legalized in one Australian state. This issue was reflected in several of the Canadian and US respondents’ discussions, whereby they had either worked with clients who had pursued assisted dying, or mentioned being open to working with these clients. Only one Australian respondent mentioned assisted dying but felt that the issue was going to be of increasing importance in shaping doula practice.

And I think going forward we’ve just had … one of our states here in Australia has made medically assisted dying legal. So I have a feeling over, say the next 12 months, there’ll be a lot more training that’s happening in regards to medically assisted dying and the role of doula in that. (T3)

Conversely, while multiple legislative attempts have been made in the United Kingdom over the last decade, assisted dying currently remains illegal, and the one UK respondent who brought up the topic was clear that EOLDs should not have this topic as a point of discussion with clients.

Finally, there are jurisdictional differences between each country regarding after-death care, which may have also shaped some respondents’ perspectives of whether these services are within EOLD scope of practice. While it is legal for the family to care for their own dead in all the countries of this study, being paid to do so remains a murky area as it can be construed as practicing funeral directing without a license. For example, in Canada, it is illegal for EOLDs, if they are acting in a professional (i.e. paid) capacity to provide any hands-on after-death care, although they can provide information to the family about how to wash, clothe, and/or move the body.^13^ In the United States, many EOLD training programs and associational bodies currently recommend not taking payment for hands-on after-death care whether or not the practitioner has training or experience as a home funeral guide. These cautions reflected many respondents’ perception that while immaculate after-death care skills are necessary, there are also fairly rigid gatekeeping attempts to keep these tasks under the auspices of funeral industry expertise. While after-death care regulations are somewhat more liberal in Australia and the United Kingdom, four of the five Australian participants mentioned growing public concern and debate about corporate concentration within the funeral industry, as well as predatory pricing and marketing, as potentially fueling interest in hiring EOLDs. Rawlings and colleagues’^2^ Australian survey found that over two thirds of respondents reported providing after-death services; however, their findings should be interpreted with caution as physical care of the body was not specified, and half of the respondents also stated that they had never been paid as a doula.

## Discussion

The results of our study evidence the enormous diversity with which the EOLD role is conceptualized and enacted, both by community-based end-of-life care practitioners who identify as an EOLD and by those who do not (or solely) identify as an EOLD. At the same time, we found considerable overlap and consistency within role description and services provided when interviews were taken as a whole. In this section, we situate our findings within the literature on the professionalization of caregiving to discuss (1) the significance of nomenclature, (2) role overlap and boundary blurring between professional and ‘supportive’ services, (3) explicit versus tacit knowledge, and (4) the importance of geographic and regional contexts as the EOLD movement develops.

Overall, half of our respondents did not identify (or solely identify) with the EOLD title, even as they reported philosophical and practice similarities to those who did. The diversity of nomenclatures used evidences that identification with specific and often idiosyncratic titles was important for many as a way to demarcate their role, philosophy of care, and specific practices. This importance of naming is highlighted in the recent legal case of a Canadian practitioner who went all the way to the Supreme Court to be able to continue using the title ‘death midwife’, which had been challenged by the Canadian Midwives Association.^13^ It also evidences that for many (approximately half of our sample), the name they choose to give their work is fluid, dependent on the specific context within which it is used. Our analysis supports Rawlings and colleagues’^14^ findings that the term ‘doula’ can be seen as ‘diminishing and restrictive’, as well as their consideration that heterogeneity of titles may also offer challenges as many in the EOLD movement seek to gain public recognition and health care integration.

At the same time, the descriptor EOLD was used significantly more than any other title by our respondents, and the increasing popularity of the term may indicate the development of a linguistic ‘boundary object’. Boundary objects are representational forms that possess interpretive flexibility, generating a shared language and classification system across multiple groups, while being robust enough to be variously defined and employed by those different actors.^20,21^ As a boundary object, the term ‘end-of-life doula’ may be useful to describe and organize the role among diverse stakeholders and across care contexts, both differentiating from and integrating with existing organizational, professional, and disciplinary boundaries in end-of-life care.

One of the main findings of our study was participants’ dual understanding of the importance of role flexibility along with an awareness of—and negotiation strategies to address—the challenges of ‘boundary blurring’. The role flexibility of EOLDs both borrows and differentiates from existing community, professional, religious, and health care roles, while also offering coordination of all these existing roles for clients and families. This mutability was simultaneously situated as one of the biggest benefits and one of the biggest challenges of providing community-based end-of-life and after-death care. Many respondents identified the need to be cognizant of and negotiate the boundaries between generalized support and professional services, and this issue was seen as a particular challenge in developing working relationships within hospice and palliative care. For some, the way to address these concerns was through ‘boundary work’,^22^ consciously demarcating and recreating divisions between fields of expertise.

Yet discussions of role flexibility and boundaries of practice were also shaped by equal emphasis on two different types of knowledge underpinning the EOLD role: explicit knowledge and tacit knowledge. Explicit knowledge can be articulated, codified, and easily transferred, such as practice competencies and scope of practice guidelines. Many participants discussed how their practices, and practice boundaries, have been shaped by formal educational and professional experiences in health and social care, whether they joined or remained affiliated with professional regulatory bodies (e.g. nursing, social work, midwifery, counseling). This included training in nonallopathic medicine and treatments (e.g. Chinese medicine, Reiki, tapping), some of which also employ tacit forms of knowledge on the basis of personal experience, emotions, and intuition. Participants also articulated the importance of extensive life experiences and/or embodied forms of knowledge (e.g. participation in the natural birth movement, informal end-of-life caregiving) necessary to role ‘maturity’ and for developing ‘innate wisdom and skills’.^2,3^ This combination of formal codified knowledge and experiential embodied knowledge was seen as enabling holistic care and key to developing therapeutic relationships, as has been evidenced elsewhere.^13^ However, for our interview participants, this hybridization was also understood as complicating the ability to clearly demarcate between which services are to be provided as part of the EOLD role, which are provided under the auspice of other formal training and/or regulations, and those based on the individual practitioner’s personal expertise and interests. Consequently, while respondents expressed significant concerns to not transgress professional role boundaries, they also referenced the value of practitioners’ experiential knowledge in self-determining these boundaries, as well as the unique needs and desires of clients within individual therapeutic relationships. The often ‘both/and’ of responses evidence that while the boundaries of formalized knowledge are key to maintaining practice boundaries of the EOLD role, so are the more tacit, experiential, and intuitive ways of knowing that trouble these boundaries. As a project of reclamation, community-based end-of-life and death care challenges the monopolization of expertise, troubling clear divisions between codified/expert and tacit/lay knowledge. However, the emergence of EOLDs also evidences a nascent demarcation of a new form of expertise, a paradox of which many participants were acutely aware.

Finally, the majority of our respondents had experience training, working with and/or attending conferences with other practitioners and educators at national and international levels. Unlike Rawlings and colleagues’^2^ findings where 40% of survey respondents were unclear about what services other doulas provided, the overwhelming majority of our participants were able to outline different practices and training models, both regionally and some even internationally. While this may be due to the level of experience of our respondents, it also evidences that the EOLD movement has significant national and international networks. Participants also discussed a range of socioeconomic conditions that have influenced the development of the EOLD role within their respective countries, as well as internationally. However, as the doula movement continues to grow, it is important to situate it within geographic and jurisdictional differences as well as similarities. In relation to similarities, the connection between the hospice and palliative care landscape in the global North and the development of EOLDs bears further research, and we are currently exploring why countries which have among the best palliative care coverage worldwide are also locations for the strongest emergence of EOLDs. On the other hand, as assisted dying legislation becomes increasingly common in the global North, yet with national and international variability, there is a need to better understand the ways in which EOLDs are/will be employed by clients desiring this form of life-ending care, and how it is/will shape similarities or differences in practices within each jurisdiction. The differential impact of funeral industry regulations on the development of EOLDs is also an important area for future research.

Overall, we do not, as Rawlings and colleagues^2^ have done, interpret the diversity—and even discord—in conceptualizing the end-of-life/death doula role and attendant services as a sign of ‘generalized confusion within the industry’. Rather, our analysis indicates that practitioners, trainers, and educators were aware of heterogeneous philosophies and practices, and were often self-reflexive about their own ambivalences regarding this diversity. Participants appeared to simultaneously celebrate practice diversity as a foundational part of their heritage while framing the challenges that this diversity brings as a common developmental ambivalence often found within the standardization and professionalization of other care roles, most notably birth doulas and midwifery.^23,24^ Other professions that bear comparison are nursing,^25^ allied health,^26^ complementary alternative medicine,^27^ social work,^28^ funeral industries,^29^ and even hospice and palliative care.^26,27^ As the EOLD movement continues to develop, and many practitioners seek recognition and integration with formal health systems, further comparison with these professional care roles is warranted. This includes considering how practitioners within existing health care and death service industries perceive the overlap of EOLDs with their own practices, and/or any negative perceptions of this ‘unregulated’ profession.^15^ For some, EOLDs offer a promising way to ‘suture’ the current division of health and death care which have been enshrined within end-of-life care in the global North. At the same time, it remains important not to prematurely foreclose inquiry into how EOLDs may continue to develop alongside, but separate from, formal bureaucratic frameworks of professionalized care in the global North. This diversity of potential future developmental pathways makes EOLDs a robust field for continued study.

## Conclusion

Contemporary concerns about the end of life within the global North are driven by health care system restructuring; changing epidemiological, demographic, and social trends; ideologies of choice, autonomy, and person-centered holistic care; and the desires of individuals, families, and communities to demedicalized dying. EOLDs evidence a new response to these complexities of modern dying. Our findings contribute substantially new information to the small body of empirical research about the EOLD role and their practices of care, and are the first research to employ an international comparative perspective. On the micro-level, our findings offer a current ‘snapshot’ of their work as it continues to evolve, and situates the diversity of approaches not merely as a limitation to be eradicated but also as a self-reflexive and foundational component of practice. On the meso-level, findings provide the first detailed taxonomy of the EOLD role and specific services on the basis of the perspective of subject experts in four countries, thereby strengthening the collaborative capacity and integration potential between diverse stakeholders and health care settings. On the macro-level, findings enable health care systems, professional associations, and policy makers to better understand the development of a new hybrid community-entrepreneurial social movement that both builds on, and differentiates from, conventional approaches to end-of-life care.

## Notes

1. We broadly define community-based end-of-life care as examples of ‘unregulated care providers’^30^ or ‘community or lay health workers’;^31^ individuals who specialize (with or without previous or formal training) in providing services, support, and care focused on decline, dying, and death within a variety of care settings, either for payment or volunteered, who are not licensed or regulated by a regulatory/professional body.

2. Mitchell, in her 2019 study of eight death doulas in Saskatchewan used themes of the following: Supportive Role, Continuous Presence, Nonmedical, Empowerment, Education and Advocacy, and Community Building.

3. Others have grouped these as predeath services, active dying services, and postdeath services.^13^ While we appreciate the ‘clean’ delineation this framework offers, it may also obscure the ways in which end-of-life doula (EOLD) services are employed across discrete events/times, such as emotional support.^32,33^
