# Erratum to “Describing the end-of-life doula role and practices of care: perspectives from four countries”

**DOI:** 10.1177/2632352421992226

**Published:** 2021-02-10

Krawczyk M and Rush M. Describing the end-of-life doula role and practices of care: perspectives from four countries. *Palliative Care and Social Practice* 2020; 14: 1-15. doi:10.1177/2632352420973226

While processing this paper, a few of the reference citations were listed incorrectly.

“Discussion” section, on page 13, column 1, paragraph 1, line 17, the correct sentence reads as, “Other professions that bear comparison are nursing,^25^ allied health,^26^ complementary alternative medicine,^27^ social work,^28^ funeral industries,^29^ and even hospice and palliative care.^30,31^”

On page 13, column 2, “Notes” section, point 1 correctly reads as, “We broadly define community-based end-of-life care as examples of ‘unregulated care providers’^32^ or ‘community or lay health workers’;^33^ individuals who specialize (with or without previous or formal training) in providing services, support, and care focused on decline, dying, and death within a variety of care settings, either for payment or volunteered, who are not licensed or regulated by a regulatory/professional body.”

On page 14, column 1, “Notes” section, point 3 correctly reads as, “Others have grouped these as predeath services, active dying services, and postdeath services.^13^ While we appreciate the ‘clean’ delineation this framework offers, it may also obscure the ways in which end-of-life doula (EOLD) services are employed across discrete events/times, such as emotional support.”

The reference renumbering has been corrected accordingly. The online version of the paper is also corrected.

